# Comparison of Bioactive Compounds and Antioxidant Activities in Differentially Pigmented *Cerasus humilis* Fruits

**DOI:** 10.3390/molecules28176272

**Published:** 2023-08-27

**Authors:** Rui Yang, Yan Yang, Yang Hu, Lu Yin, Pengyan Qu, Pengfei Wang, Xiaopeng Mu, Shuai Zhang, Peng Xie, Chunzhen Cheng, Jiancheng Zhang

**Affiliations:** College of Horticulture, Shanxi Agricultural University, Jinzhong 030801, China; yangrui13934147962@163.com (R.Y.); 18404968975@163.com (Y.Y.); hy16634257765@163.com (Y.H.); yl18434764709@163.com (L.Y.); yanyan5235@outlook.com (P.Q.); 13835436501@163.com (P.W.); 15110671026@163.com (X.M.); wwzs_1990@aliyun.com (S.Z.); xiepeng@sxau.edu.cn (P.X.)

**Keywords:** *Cerasus humilis*, bioactive substances, antioxidant capacity, carotenoids, flavonoids

## Abstract

Chinese dwarf cherry (*Cerasus humilis*) is a wild fruit tree and medicinal plant endemic to China. Its fruits are rich in various bioactive compounds, such as flavonoids and carotenoids, which contribute greatly to their high antioxidant capacity. In this study, the contents of bioactive substances (chlorophyll, carotenoids, ascorbic acid, anthocyanin, total flavonoids, and total phenols), antioxidant capacities, 2,2-diphenyl-1-picrylhydrazyl (DPPH) and 2,2′-azino-bis (3-ethylbenzothiazoline-6-sulfonicacid) (ABTS^+^) scavenging ability, and ferric-reducing antioxidant power (FRAP)) in differentially pigmented *C. humilis* fruits of four varieties were determined and compared. The results revealed that anthocyanin, total flavonoids and total phenols were the three main components responsible for the antioxidant activity of *C*. *humilis* fruits. ‘Jinou No.1’ fruits with dark red peel and red flesh had the highest contents of anthocyanin, total flavonoids, and total phenols, as well as the highest antioxidant capacities; ‘Nongda No.5’ fruits with yellow-green peel and yellow flesh had the highest contents of carotenoids and chlorophyll, while ‘Nongda No.6’ fruit had the highest ascorbic acid content. To further reveal the molecular mechanism underlying differences in the accumulation of carotenoids and flavonoids among differentially pigmented *C. humilis* fruits, the expression patterns of structural genes involved in the biosynthesis of the two compounds were investigated. Correlation analysis results revealed that the content of carotenoids in *C. humilis* fruits was very significantly positively correlated with the expression of the *ChCHYB*, *ChZEP*, *ChVDE*, *ChNSY*, *ChCCD1*, *ChCCD4*, *ChNCED1*, and *ChNCED5* genes (*p* < 0.01) and significantly negatively correlated with the expression of *ChZDS* (*p* < 0.05). The anthocyanin content was very significantly positively correlated with *ChCHS*, *ChFLS*, and *ChUFGT* expression (*p* < 0.01). The total flavonoid content was very significantly positively correlated with the expression of *ChCHS*, *ChUFGT*, and *ChC4H* (*p* < 0.01) and significantly positively correlated with *ChFLS* expression (*p* < 0.05). This study can provide a basis for understanding the differences in the accumulation of bioactive substances, and is helpful for clarifying the mechanisms underlying the accumulation of various carotenoids and flavonoids among differentially pigmented *C. humilis* fruits.

## 1. Introduction

Oxidative stress caused by free radical accumulation is very harmful to the human immune system [1]. Accumulated evidence has revealed that carotenoids (including α-carotene, β-carotene, α-cryptoxanthin, and β-cryptoxanthin), chlorophylls, ascorbic acid, total phenols, and total flavonoids (including flavones, isoflavones, flavanols and anthocyanin), as well as other bioactive substances, have strong antioxidant capacities [2,3,4,5]. Therefore, these bioactive substances are regarded as important sources of new green therapeutic natural compounds [6]. For example, carotenoids are natural pigments beneficial to the eyes and cardiovascular system [7], while phenols and flavonoids have been widely identified as free radical scavenging molecules [1,8]. Moreover, anthocyanin is a water-soluble flavonoid that has been considered a hotspot in health-beneficial compounds research in recent years [9].

The Chinese dwarf cherry (*Cerasus humilis* or *Prunus humilis*) is a China-endemic wild fruit tree and medicinal plant belonging to the Rosaceae family [10,11]. Its fruit kernel, known as ‘Yuliren’, has long been used in traditional Chinese medicine [12]. Its fruits can be eaten freshly or processed into dried fruits, vinegar, wine, juice, and jam. Moreover, they can be used as important raw materials in the production of healthcare products [13]. *C. humilis* fruits are rich in carotenoids, flavonoids, phenols, and other bioactive substances [14]. Due to their strong antioxidant capacity, *C. humilis* fruits have great potential to be applied in the healthcare industry. *C. humilis* polyphenols can reduce obesity and lower blood lipid and glucose levels by downregulating the expression of key transcription factors (*PPARγ* and *C/EBPα*), thereby inhibiting adipocyte differentiation and accelerating glucose and fat metabolism [11]. Fermented *C. humilis* fruit juice can increase the expression of immune protein-related genes, thereby regulating intestinal mucosal immune function and maintaining intestinal mucosal barrier homeostasis [15,16]. Additionally, fermented *C. humilis* fruits can activate the antioxidase system, ameliorate hyperlipidemia and cholesterol over-accumulation, and restore damage due to abnormalities in antioxidant and lipid metabolism caused by hyperlipidemia [17].

China is rich in *C. humilis* varieties and resources, with the main distribution and cultivation areas located in Shanxi, Shandong, Hebei, Inner Mongolia, Heilongjiang and Liaoning provinces [11]. In view of the functions and beneficial health effects of the bioactive substances in *C. humilis* fruits, it is necessary to carry out determination and comparative study of *C. humilis* germplasm resources. In this study, the contents of chlorophyll, carotenoids, ascorbic acid, anthocyanin, total flavonoids, and total phenols, as well as the antioxidant capacities of ABTS^+^, DPPH, and FRAP in differentially pigmented fruits of four main *C. humilis* varieties from Shanxi Province, China were determined and compared. Additionally, based on the expression analysis of biosynthetic structural genes, key factors affecting the accumulation of carotenoids and flavonoids in *C. humilis* fruits were uncovered. This study provides a foundation for revealing the bioactive substance contents and differences in antioxidant capacity among fruits of different *C. humilis* varieties and a scientific basis for the future utilization of differently pigmented *C. humilis* fruits.

## 2. Results

### 2.1. Fruit Appearance Quality Comparision Results of the Four C. humilis Varieties

**Figure 1:**
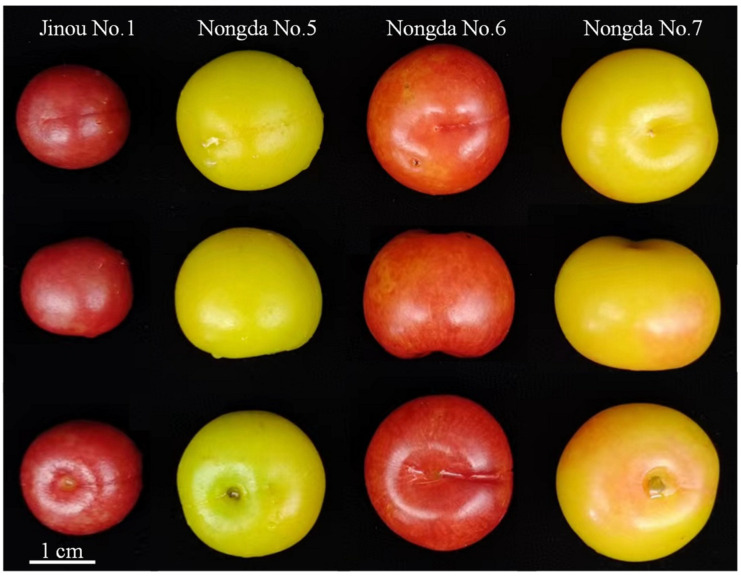
Mature fruits of the four *C*. *humilis* varieties used in this study.

The external fruit qualities of the four *C. humilis* varieties were first observed. The fruit of ‘Jinou No.1’ was the smallest among the four *C. humilis* varieties. Its fruit is oblate spheroidal with dark red peel. The fruit of ‘Nongda No.5’ is spheroidal and its peel is yellow-green. The fruit of ‘Nongda No.6’ is nearly spherical with red peel. The fruit of ‘Nongda No.7’ is oblate spheroidal and its peel is yellow with slight flush (Figure 1). With the exception of the flesh of ‘Jinou No.1’, which is red, the flesh colors of other *C. humilis* fruits are all yellow. The fruit color index is one an important index for fruit quality evaluation [18]. By measuring the fruit color index of mature fruits of all four varieties, it was found that the L* value of ‘Nongda No.5’ was the highest (suggesting that its fruit peel is the brightest), followed successively by ‘Nongda No.7’, ‘Nongda No.6’, and ‘Jinou No.1’. Among the four *C. humilis* fruits, the a* value of ‘Nongda No.5’ was less than 0. The b* value of ‘Nongda No.5’ was the highest (indicating that its fruit peel was the yellowest), followed by ‘Nongda No.7’, ‘Nongda No.6’, and ‘Jinou No.1’ (Table 1).

### 2.2. Determination Results of Bioactive Substance Contents in Fruits of Four C. humilis Varieties

The contents of bioactive substances in fruits of the four *C. humilis* varieties were measured. The results showed that the chlorophyll and carotenoid contents in ‘Nongda No. 5’ fruits were both the highest. The chlorophyll content in ‘Nongda No.5’ fruit was approximately 2.38-, 2.17-, and 2.66-fold higher than those of ‘Jinou No.1’, ‘Nongda No.6’, and ‘Nongda No.7’, respectively. Its content of carotenoids was 2.18-, 2.70-, and 2.98-fold higher than those of ‘Jinou No.1’, ‘Nongda No.6’, and ‘Nongda No.7’, respectively (Table 1).

The ascorbic acid content in ‘Nongda No.6’ fruit was the highest (76.10 ± 8.64 mg/100 g) among the four *C. humilis* verities (Table 1), at 1.07-, 1.20-, and 2.29-fold higher than those in ‘Nongda No.5’, ‘Nongda No.7’, and ‘Jinou No.1’ fruit, respectively. Moreover, the ascorbic acid content in ‘Jinou No.1’ fruits was significantly lower than in the other three varieties (*p* < 0.05), accounting for only 43.59%, 46.42%, and 52.36% of ‘Nongda No.6’, ‘Nongda No.5’, and ‘Nongda No.7’, respectively.

Among the fruits of the four *C. humilis* varieties, the contents of total phenols, total flavonoids, and anthocyanin in ‘Jinou No.1’ fruit were all the highest. Its total content of phenols (8.50 ± 0.38 mg/g) was 4.86-, 6.03-, and 6.49-fold higher than those of ‘Nongda No.7’, ‘Nongda No.6’, and ‘Nongda No.5’, respectively. Its total content of flavonoids (0.58 ± 0.04 mg/g) was 1.53-, 1.53-, and 6.44-fold higher than those of ‘Nongda No.6’ (0.38 ± 0.03 mg/g), ‘Nongda No.7’ (0.38 ± 0.06 mg/g), and ‘Nongda No.5’ (0.09 ± 0.02 mg/g), respectively. Its anthocyanin content (16.66 ± 0.29 mg/g) was 38.74-, 1.95-, and 5.85-fold higher than those of ‘Nongda No.5’, ‘Nongda No.6’, and ‘Nongda No.7’ fruit, respectively (Table 1).

### 2.3. Comparison of Antioxidant Capacities in Fruits of Four Different C. humilis Varieties

The FRAP, ABTS^+^, and DPPH free radical scavenging abilities of ‘Jinou No.1′ fruit were all significantly higher than fruits of the other three varieties (*p* < 0.05) (Table 1). The FRAP (2227.28 ± 277.55 mg TE/kg FW) of ‘Jinou No.1’ fruit was 1.82-, 2.1-, and 2.41-fold higher than those of ‘Nongda No.6’, ‘Nongda No.7’, and ‘Nongda No.5’, respectively. The ABTS^+^ free radical scavenging ability of ‘Jinou No.1’ fruit was 1.91-, 1.91-, and 2.03-fold higher than those of ‘Nongda No.7’, ‘Nongda No.6’, and ‘Nongda No.5’, respectively. The DPPH free radical scavenging ability of ‘Jinou No.1’ was significantly higher than those of ‘Nongda No.7’ and ‘Nongda No.6’ (*p* < 0.05) and slightly higher than that of ‘Nongda No.5’.

### 2.4. Correlation and Principal Component Analysis (PCA) of Bioactive Substance Contents and Antioxidant Capacities

PCA of the bioactive substance contents and antioxidant capacities of fruits of the four different *C. humilis* varieties was performed (Table 2). The anthocyanin content was found to be very significant positively correlated with the total flavonoid content, total phenol content, FRAP, and ABTS^+^ (*p* < 0.01). The total phenol content was very significantly positively correlated with DPPH (*p* < 0.01). There were significant correlations among other parameters as well (*p* < 0.05). For example, very significant positive correlations were found among the contents of chlorophyll, chlorophyll a, chlorophyll b, and carotenoids (*p* < 0.01); the ascorbic acid content was very significantly negatively correlated with anthocyanin content, total flavonoid content, total phenol content, FRAP, ABTS^+^, and DPPH (*p* < 0.01); and ABTS^+^ was very significantly positively correlated with FRAP and DPPH (*p* < 0.01).

**Figure 2:**
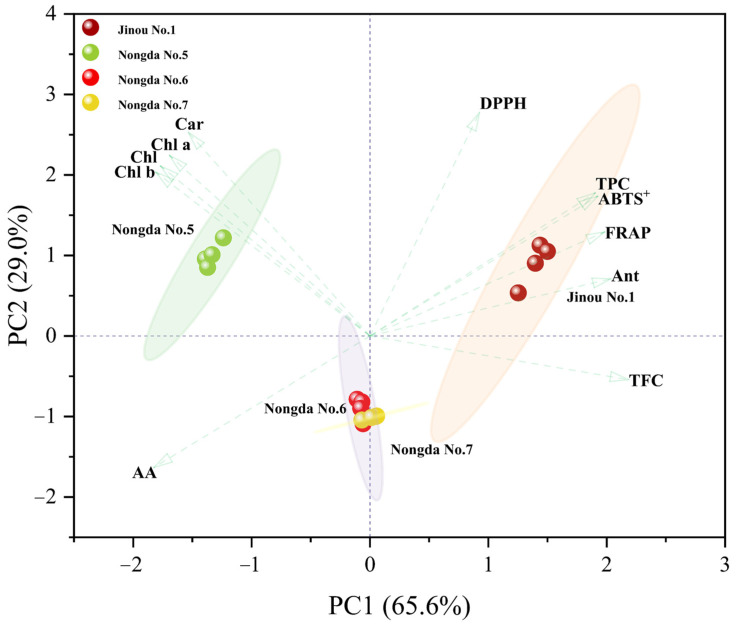
PCA results for the fruit parameters of the four different *C. humilis* varieties. Chl: Chlorophyll; Chl a: chlorophyll a; Chl b: chlorophyll b; Car: carotenoids; ABA: abscisic acid; Ant: anthocyanin; TFC: total flavonoids; TPC: total phenols; AA: ascorbic acid; FRAP: ferric reducing antioxidant power; ABTS^+^: 2,2′-azino-bis (3-ethylbenzothiazoline-6-sulfonic acid) radical cation scavenging ability; DPPH: 2,2-diphenyl-1-picrylhydrazyl free radical scavenging ability.

Our PCA results revealed that the contribution rates of the first and second principal components (PC1 and PC2) were 65.6 % and 29.0 % (Figure 2), respectively, indicating that they covered the comprehensive information of most parameters. ‘Jinou No.1’, with the highest contents of anthocyanin, total flavonoids, and total phenols as well as the strongest antioxidant capacities, scored the highest in PC1, while ‘Nongda No.5’, with the highest carotenoids and chlorophyll contents, scored the highest in PC2.

### 2.5. Expression Analysis of Carotenoid Biosynthesis-Related Genes in Fruits of Four C. humilis Varieties

**Figure 3:**
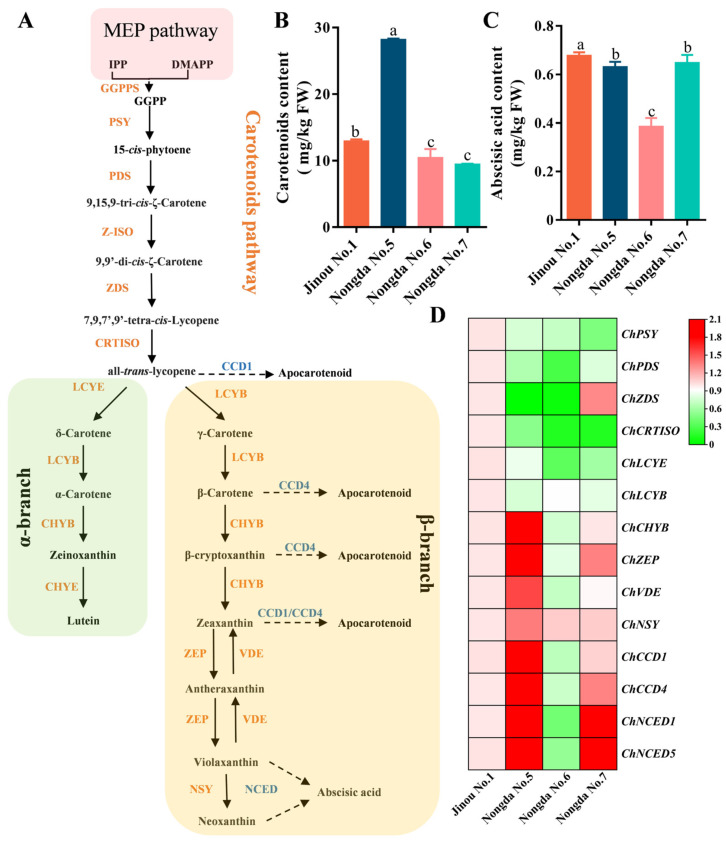
Carotenoid metabolism comparison in fruits of the four different *C*. *humilis* varieties: (**A**) carotenoid synthesis and metabolic pathways; (**B**) comparison of carotenoid contents in four different *C*. *humilis* varieties; (**C**) comparison of ABA content in four different *C*. *humilis* varieties; (**D**) expression heatmap for carotenoid biosynthesis-related structural genes. MEP: 2-C-methyl-D-erythritol-4-phosphate; IPP: isopentenyl diphosphate; DMAPP: dimethylallyl diphosphate; GGPP: geranylgeranyl diphosphate; PSY: phytoene synthase; PDS: phytoene desaturase; Z-ISO: ζ-carotene isomerase; ZDS: ζ-carotene desaturase; CRTISO: carotene isomerase; LCYB: Lycopene β-cyclase; LCYE: lycopene ε-cyclase; CHYB: β-carotene hydroxylase; CHYE: ε-carotene hydroxylase; ZEP: zeaxanthin epoxidase; VDE: violaxanthin de-epoxidase; NSY: neoxanthin synthase; CCD: carotenoid cleavage dioxygenase; NCED: 9-*cis*-epoxycarotenoid dioxygenase. The different letters above the columns in (**B**,**C**) indicate significant differences at the *p* < 0.05 level.

To explore the mechanisms of the differentially accumulated carotenoids in fruits of different *C. humilis* varieties, quantitative real time PCR (qRT-PCR) was used to compare the expression of ten genes related to carotenoid biosynthesis (*ChPSY*, *ChPDS*, *ChZDS*, *ChCRTISO*, *ChLCYE*, *ChLCYB*, *ChCHYB*, *ChZEP*, *ChVDE*, and *ChNSY*) and four genes related to carotenoid degradation (*ChCCD1*, *ChCCD4*, *ChNCED1*, and *ChNCED5*). Meanwhile, the ABA contents in *C. humilis* fruits were determined as well (Figure 3A–C). The results showed that the expression levels of *ChPSY*, *ChPDS*, *ChCRTISO*, *ChLCYE*, and *ChLCYB* in ‘Jinou No.1’ fruit were the highest. *ChZDS* was expressed the highest in ‘Nongda No.7’. Except for *ChPSY*, *ChLCYB*, and *ChNSY*, the expression levels of other genes were the lowest in ‘Nongda No.6’. The expression levels of the *ChCHYB*, *ChZEP*, *ChVDE*, and *ChNSY* genes related to carotenoid biosynthesis were the highest in ‘Nongda No.5’, while the expression levels of the *ChCCD1*, *ChCCD4*, *ChNCED1*, and *ChNCED5* genes related to carotenoid degradation were the highest in ‘Nongda No.5’ and the lowest in ‘Nongda No.6’ (Figure 3D).

**Figure 4:**
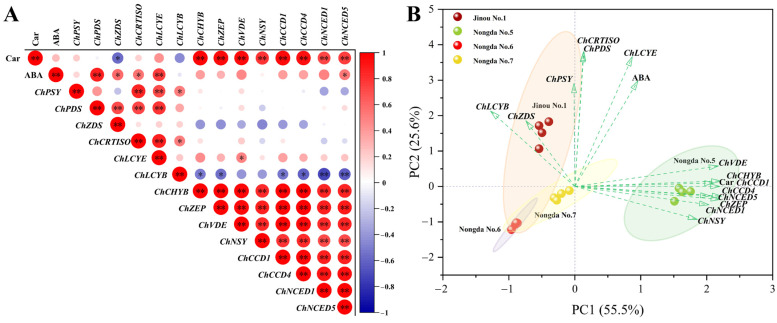
Correlation (**A**) and PCA (**B**) analysis results for the of carotenoid and ABA contents and the expression levels of carotenoid metabolism-related genes. Car: carotenoids; ABA: abscisic acid. * and ** indicate significant correlation (*p* < 0.05) and very significant correlation (*p* < 0.01), respectively.

The correlations among carotenoid and ABA contents and the expression levels of carotenoid metabolism-related genes in fruits of different *C. humilis* varieties were further analyzed (Figure 4A). The results show that the content of carotenoids was very significantly positively correlated with *ChCHYB*, *ChZEP*, *ChVDE*, *ChNSY*, *ChCCD1*, *ChCCD4*, *ChNCED1*, and *ChNCED5* (*p* < 0.01), significantly negatively correlated with *ChZDS* (*p* < 0.05), and positively correlated with ABA content. ABA content was very significantly positively correlated with *ChPDS* and *ChLCYE* (*p* < 0.01) and significantly positively correlated with *ChZDS*, *ChCRTISO*, and *ChNCED5* (*p* < 0.05). In addition, although the correlation was not significant, ABA content was positively correlated with the expression levels of *ChCCD1*, *ChCCD4*, and *ChNCED1*.

PCA based on the contents of carotenoids and ABA and expression of carotenoid metabolism-related genes show that the four *C. humilis* varieties can be clearly separated; the biological replicates of each variety were closely clustered and located within the 95% confidence interval (Figure 4B). The contribution rates of PC1 and PC2 were 55.5% and 25.6%, respectively, and the cumulative contribution rate of the two principal components accounted for 81.1%. It is worth noting that ‘Nongda No.5’, with the highest carotenoid content, scored the highest in PC1, while ‘Jinou No.1’, with the highest ABA content and the highest expression of *ChPSY*, *ChPDS*, *ChCRTISO*, *ChLCYE,* and *ChLCYB* genes, scored the highest in PC2. ‘Nongda No.6’, with the lowest ABA content, scored the lowest in both PC1 and PC2.

### 2.6. Expression Analysis of Flavonoid Metabolism Related Genes in Fruits of Four C. humilis Varieties

**Figure 5:**
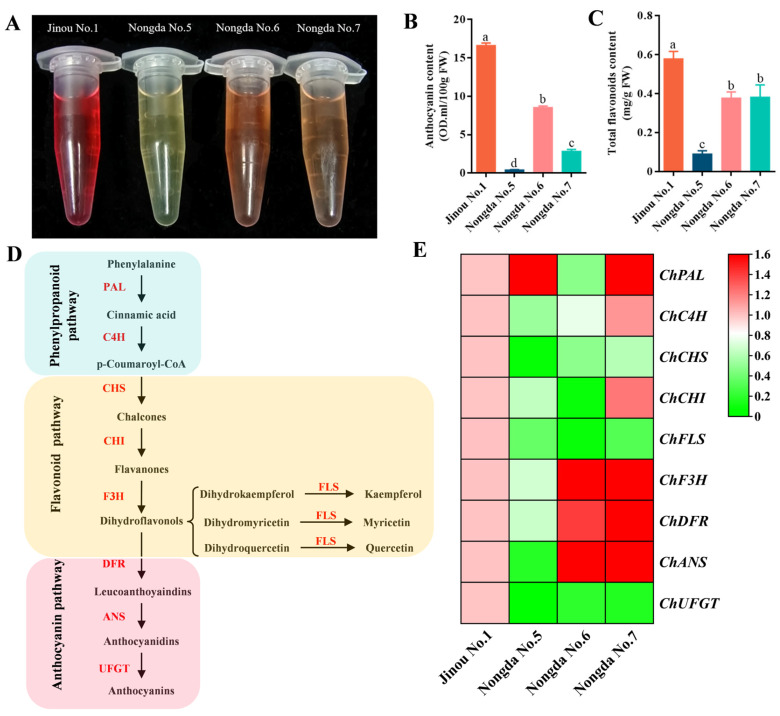
Fruit flavonoid metabolism comparison of the four different *C*. *humilis* varieties. (**A**) Anthocyanin extract solution of *C*. *humilis* fruits. The color of anthocyanin extract of ‘Jinou No.1’ was the reddest, the anthocyanin extract of ‘Nongda No.5’ was yellow-green, the anthocyanin extract of ‘Nongda No.6’ and ‘Nongda No.7’ was orange, and the color of ‘Nongda No.6’ was slightly darker than that of ‘Nongda No.7’. (**B**) Anthocyanin contents in fruits of four *C*. *humilis* varieties: (**C**) total flavonoid content in fruits of four *C*. *humilis* varieties; (**D**) flavonoid biosynthesis pathway; (**E**) expression heatmap for flavonoid biosynthesis-related structural genes. PAL: phenylalanine ammonia lyase; C4H: cinnamate 4-hydroxylase; CHS: chalcone synthase; CHI: chalcone isomerase; FLS: flavonol synthase; F3H: flavanone 3-hydroxylase; DFR: dihydroflavonol 4-reductase; ANS: anthocyanin synthase; UFGT: UDP-glucose: flavonoid 3-O-glucosyltransferase. The different letters above the columns in (**B**,**C**) indicate significant differences at the *p* < 0.05 level.

In order to reveal the mechanism underlying the differences in flavonoid accumulation among fruits of the four *C. humilis* varieties, the expression of flavonoid metabolism-related genes was quantitatively verified (Figure 5). The expression levels of anthocyanin and flavonoid biosynthesis-related genes, especially *ChFLS* and *ChUFGT* were significantly higher in ‘Jinou No.1’ fruits than in the other three varieties. In fruits of ‘Nongda No.5’, all other genes except *ChPAL* were found to have low levels of expression. The expression levels of *ChF3H*, *ChDFR,* and *ChANS* in ‘Nongda No.6’ and ‘Nongda No.7’ were much higher than those in the other two varieties.

**Figure 6:**
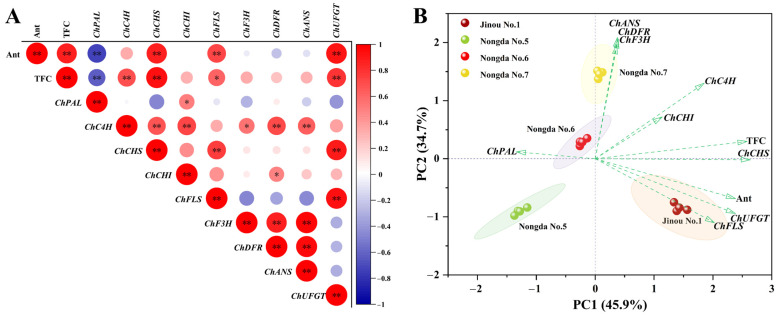
Correlation analysis (**A**) and PCA (**B**) results for the contents of anthocyanin and total flavonoids and expression levels of flavonoid metabolism-related genes. Ant: anthocyanin; TFC: total flavonoids. * and ** indicate significant correlation (*p* < 0.05) and very significant correlation (*p* < 0.01), respectively.

By analyzing the correlations among anthocyanin content, total flavonoid content, and the expression of synthesis-related genes (Figure 6A), it was found that anthocyanin content in *C. humilis* fruits was very significantly positively correlated with total flavonoid content and expression levels of the *ChCHS*, *ChFLS*, and *ChUFGT* genes (*p* < 0.01). The total flavonoid content was very significantly positively correlated with the expression of *ChC4H*, *ChCHS* and *ChUFGT* (*p* < 0.01), and significantly positively correlated with the expression of *ChFLS* (*p* < 0.05). Interestingly, *ChCHS* and *ChUFGT*, which are positively correlated with the contents of both anthocyanin and total flavonoids, were all expressed the highest in ‘Jinou No.1’ fruit and the lowest in ‘Nongda No.5’ fruit.

PCA analysis was performed based on anthocyanin and total flavonoid content and on flavonoid metabolism-related structural gene expression levels (Figure 6B). The results show that ‘Jinou No.1’, with the highest contents of anthocyanin and total flavonoids, and the highest expression levels of *ChUFGT*, *ChFLS*, and *ChCHS*, scored the highest in PC1, while ‘Nongda No.7’, with the highest expression levels of the *ChC4H*, *ChCHI*, *ChF3H*, *ChDFR*, and *ChANS* genes, scored the highest in PC2 and ‘Nongda No.5’, with the lowest anthocyanin and total flavonoids contents, scored the lowest in both PC1 and PC2.

## 3. Discussion

### 3.1. The Bioactive Substance Contents and Antioxidant Capacities of Differently Pigmented C. humilis Fruits Vary Greatly

In this study, significant differences in the bioactive substance contents and DPPH, ABTS^+^, and FRAP antioxidant capacities of four differentially pigmented *C. humilis* fruits were discovered. In onions, the contents of anthocyanin, total flavonoids and total phenols, and antioxidant capacity of red onions have been found to be higher than those of yellow and white onions, indicating that darker colors are related to higher contents of anthocyanin, total flavonoids, and total phenols as well as to stronger antioxidant capacity [19]. Consistently, in this study we found that ‘Jinou No.1’ fruit had the highest anthocyanin, total flavonoid, and total phenol contents and the strongest antioxidant capacity. Interestingly, the ascorbic acid content in fruits of ‘Nongda No.6’, ‘Nongda No.5’, and ‘Nongda No.7’ was significantly higher than in ‘Jinou No.1’ fruits, indicating that these are more suitable for use as a natural source of ascorbic acid [20]. Additionally, our correlation analysis results reveal that the ascorbic acid content in *C. humilis* fruits is very significantly negatively correlated with anthocyanin content, total flavonoid content, total phenol content, FRAP, ABTS^+^, and DPPH (*p* < 0.01).

Carotenoids and chlorophyll are important pigments, respectively conferring yellow and green colors on fruits. They both have strong antioxidant and healthcare values [21,22]. In this study, it was found that the contents of carotenoids and chlorophyll in ‘Nongda No.5’ fruits were more than twice of those of the other three varieties, indicating that this variety is rich in carotenoids and chlorophyll.

Phenols are highly beneficial in terms of their health values [23,24,25]. *C. humilis* fruits are rich in total flavonoids and phenols [13]. Among the four varieties, the highest amounts of total flavonoids and phenols and strongest antioxidant capacity were identified in ’Jinou No.1’ fruits, suggesting that this variety might have great potential for use as a raw material in producing bioactive substances for clinical researches. 

### 3.2. Anthocyanin, Total Flavonoids, and Total Phenols Are the Three Main Components Affecting the Antioxidant Activity of C. humilis Fruits

The antioxidant activity of fruits is mainly dependent on the accumulation of bioactive substances such as anthocyanin, total flavonoids, and total phenols [26,27]. In fruits of most plants, the contents of total flavonoids and total phenols are reported to be positively correlated with the antioxidant capacity [28]. Anthocyanin has free radical scavenging activity and can reduce oxidative stress [29]. The total flavonoids, total phenols, and antioxidant capacities (DPPH, ABTS^+^, and FRAP) of thyme have been shown to be positively correlated [30]. The antioxidant capacity of pomegranate was shown to be significantly positively correlated with the contents of anthocyanin, total flavonoids and total phenols [31]. The of total flavonoid and total phenol contents in grapes have been positively correlated with antioxidant capacity (ABTS^+^ and FRAP) [32]. In this study, the contents of anthocyanin, total flavonoids, and total phenols in the fruit of ‘Jinou No.1’ were found to be higher than in the other three varieties, and its antioxidant capacity was the highest. Moreover, we found that the contents of anthocyanin, total flavonoids, and total phenols in *C. humilis* fruits were positively correlated with antioxidant capacities (DPPH, ABTS^+^, and FRAP), indicating that these three bioactive substances may synergistically regulate the antioxidant capacity of *C. humilis* fruits.

### 3.3. The Accumulation of Carotenoids in Fruits of Different C. humilis Varieties Are Closely Related to the Expression of Carotenoid Metabolism-Related Genes

Gene expression analysis revealed that the expression levels of carotenoid biosynthesis-related genes (*ChCHYB*, *ChZEP*, *ChVDE*, and *ChNSY*) and carotenoid degradation-related genes (*ChCCD1*, *ChCCD4*, *ChNCED1*, and *ChNCED5*) in ‘Nongda No.5’ fruits (with high carotenoid content) were significantly higher than other three varieties. The ABA content in fruits of ‘Nongda No.6’ was the lowest among the four *C*. *humilis* varieties. Consistently, except for *ChPSY*, *ChLCYB*, and *ChNSY*, the expression levels of other carotenoid metabolism-related genes in ‘Nongda No.6’ were all the lowest. Correlation analysis showed that carotenoid content was very significantly positively correlated with expression of *ChCHYB*, *ChZEP*, *ChVDE*, *ChNSY*, *ChCCD1*, *ChCCD4*, *ChNCED1*, and *ChNCED5* (*p* < 0.01), significantly negatively correlated with *ChZDS* expression (*p* < 0.05), and positively correlated with ABA content.

PSY is the first key rate-limiting enzyme in the carotenoid biosynthesis pathway [33]. Overexpression of *PSY* in maize callus has been found to significantly increase the accumulation of carotenoids (*p* < 0.05) [34]. In our study, we found that the carotenoid content in *C. humilis* fruits was positively correlated with the expression level of *ChPSY*. Moreover, the expression level of *ChPSY* in ‘Nongda No.7’ (with the lowest carotenoid content among the four *C. humilis* varieties) was found to be the lowest.

ChCCD1 has been identified as playing a key role in the degradation of carotenoids [35]. In our study, we found that carotenoid content was significantly positively correlated with the expression levels of *ChCCD1* and *ChCCD4* (*p* < 0.05). Nine-*cis*-epoxycarotenoid dioxygenase (NCED) is a key enzyme connecting the carotenoid degradation and ABA biosynthesis pathways [36]. ABA content has consistently been found to be correlated to the expression level of *NCED* [37]. It has been reported that the expression level of *IbNCED3* is positively correlated with the total content of carotenoids in the SS8 sweet potato variety [38]. In this study, the highest expression level of *ChNCED* and high ABA content were consistently found in ‘Nongda No.5’ (with the highest carotenoid content), while the lowest expression level of *ChNCED* and lowest ABA content was found in ‘Nongda No.6’ fruit, which has a low content of carotenoids.

### 3.4. The Expression of Flavonoid Biosynthesis-Related Genes Such as ChCHS, ChUFGT, and ChFLS Is Very Significantly or Significantly Positively Correlated with Flavonoid Content in C. humilis Fruits

Chalcone synthase (CHS) catalyzes the first step of flavonoid biosynthesis [39]. The expression of *CHS* has been shown to be very significantly positively correlated with of anthocyanin and flavonoid contents [40]. In strawberry, an increase in *CHS* expression level and the accumulation of anthocyanin and flavonoids were found to occur simultaneously [41]. Flavonol synthase (FLS) is a key enzyme in the biosynthesis of flavonols in the flavonoid pathway [42,43], while UFGT is a key enzyme catalyzing the final step of anthocyanin biosynthesis. The anthocyanin content of myrtle berries was found to be strongly positively correlated with the expression level of *UFGT*, and the expression level of *UFGT* was the highest in dark blue fruits with high anthocyanin content [44]. In this study, the expression levels of *ChCHS*, *ChUFGT*, and *ChFLS* were found to be very significantly (*p* < 0.01) or significantly (*p* < 0.05) correlated with the contents of anthocyanin and total flavonoids in *C*. *humilis* fruits. Moreover, consistently with the finding of the highest anthocyanin and total flavonoids contents in ‘Jinou No.1’ fruits and the lowest contents in ‘Nongda No.5’ fruits, the expression levels of *ChCHS* and *ChUFGT* were the highest in ‘Jinou No.1’ fruits and the lowest in ‘Nongda No.5’ fruits. This indicates that these three genes are closely related to the biosynthesis of anthocyanin and flavonoids in *C*. *humilis* fruits.

## 4. Materials and Methods

### 4.1. Plant Materials

‘Jinou No.1’, ‘Nongda No.5’, ‘Nongda No.6’, and ‘Nongda No.7’ mature fruits with relatively uniform size and color and no mechanical or pest damage were harvested from the same *C. humilis* germplasm nursery located in Shanxi Agricultural University, then stored on ice and taken back to the laboratory. Color parameters (L*, a*, and b* values) of twenty fruits from each variety were measured using a CR8 colorimeter (3nh, Guangzhou, China) [45]. After removing the seeds, the fruits were cut into small pieces, frozen in liquid nitrogen, and stored in a refrigerator at −80 °C for further use.

### 4.2. Determination of Carotenoid, Chlorophyll, Anthocyanin, and Ascorbic Acid Contents

Extraction of carotenoids and chlorophyll and determination of their contents was carried out according to the methods of Gao et al. [46] and Zhang et al. [38]. After grinding fruits into a fine powder in liquid nitrogen, 1 g of sample was added to 5 mL of acetone (containing 0.1% butyl hydroxytoluene) and ultrasonically extracted for 60 min. Then, the supernatant was collected by centrifugation at 10,000 rpm for 15 min. A spectrophotometer (UV-1800, Shanghai Meixi Instrument Co., Ltd., Shanghai, China) was used to measure the absorbance of the supernatant at 663 nm, 645 nm, and 450 nm. Carotenoid content was calculated using the following formula: content (mg/kg) = ABS (OD) × extract volume (mL) × dilution times/sample weight (kg)/2500 (the average absorbance of 1% carotenoids at the maximum absorption wavelength). Chlorophyll content was calculated using the following formula: content (mg/kg) = (20.21 × ABS (OD_645_) + 8.02 × ABS (OD_663_)) × extract volume (mL) × dilution times/sample weight (kg)/1000.

Anthocyanin content in *C*. *humilis* fruits was extracted and determined according to the method of Zhuang et al. [47]. Briefly, 2.5 g of fruit peel was homogenized with acidified ethanol containing 85 mL of 95% ethanol and 15 mL of 1.5 mol/L hydrochloric acid per 100 mL and diluted to 25 mL with acidified ethanol. After being placed in the dark at room temperature for 24 h, this solution was centrifuged at 10,000 rpm for 15 min. The supernatant was collected and subjected to absorbance value measurement at 535 nm using a spectrophotometer (UV-1800, Shanghai Meixi Instrument Co., Ltd.). Anthocyanin content was calculated using the following formula: content (OD mL/100 g FW) = ABS (OD) × extract volume (mL) × dilution times/sample weight (g)/extinction coefficient (98.2) × 100. The content of ascorbic acid was measured using the 2,6-dichloroindophenol titration method [48].

### 4.3. Determination of Total Flavonoids, Total Phenols, and Antioxidant Capacity

After grinding fruits into fine powder in liquid nitrogen, 2.5 g of fruit sample was added to 10 mL of 80% ethanol, mixed by whirlpool oscillation, extracted by ultrasound at 40 kHz for 15 min, and centrifuged at 5000 rpm for 10 min at 4 °C. The supernatant was collected, and after two rounds of extraction the collected supernatants were pooled, diluted to 25 mL with 80% ethanol, and used to determine the contents of total flavonoids and total phenols along with the antioxidant capacity. The total flavonoid content and ABTS^+^ free radical scavenging ability of the fruits were determined by reference to the method of Fu et al. [49]. The total phenol content, DPPH free radical scavenging ability, and ferric reducing antioxidant power (FRAP) of the fruits were determined according to the method described by Clarke et al. [50].

### 4.4. Determination of Abscisic Acid (ABA) Content

The ABA contents of the four varieties of *C. humilis* fruits were determined using a plant abscisic acid enzyme-linked immunosorbent assay kit (Jiankang Biological, Shanghai, China).

### 4.5. Gene Expression Analysis

The flavonoid and carotenoid metabolism-related protein sequences of *Arabidopsis thaliana* and *Citrus sinensis* were downloaded from the *A. thaliana* genome website (https://www.arabidopsis.org/, accessed on 5 March 2023.) and Phytozome13 (https://phytozome-next.jgi.doe.gov/, accessed on 5 March 2023.), respectively. Using these as queries, BLASTP searches against the *C. humilis* protein data were performed using TBtools to identify candidate flavonoid biosynthesis-related (ChPAL, ChC4H, ChCHS, ChCHI, ChFLS, ChF3H, ChDFR, ChANS, and ChUFGT) and carotenoid metabolism-related (ChPSY, ChPDS, ChZDS, ChCRTISO, ChLCYE, ChLCYB, ChCHYB, ChZEP, ChVDE, and ChNSY) proteins of *C. humilis*. According to their coding sequences, primers were designed using Primer 3.0; the primers of the *ChCCD1*, *ChCCD4*, *ChNCED1*, and *ChNCED5* genes were synthesized according to Cheng et al. [35] (Table S1). A Trizol RNA Extraction Kit (TaKaRa, Dalian, China) was used to isolate the total RNA from the mature fruits of the four *C. humilis* cultivars. Then, high-quality RNA was used for cDNA synthesis using a PrimeScript^TM^ RT reagent Kit with a gDNA Eraser (Perfect Real Time) kit (TaKaRa, Dalian, China). qRT-PCR reactions were performed on a QuantStudio 3 (Applied Biosystems, Shanghai, China) real-time quantitative fluorescent PCR instrument using a TB Green^®^ Premix Ex Taq^TM^ II kit (Tli RNaseH Plus; TaKaRa, Dalian, China). With *ChActin* as the internal reference gene, the relative expression levels of the selected *C. humilis* genes in the fruits of the four cultivars were calculated using the 2^−∆∆Ct^ method [35]. Three biological and three technical replications were made during qRT-PCR analysis of the selected genes.

### 4.6. Data Analysis

All data are presented as the mean ± standard deviation of at least three biological repetitions. OriginPro 9.0 was used for Pearson correlation analysis and principal component analysis (PCA). One-way analysis of variance (ANOVA) in SPSS 25.0 was used for statistical analysis of the data at *p* < 0.05 and/or *p* < 0.01 levels. Figures were created using GraphPad Prism 8.0.

## 5. Conclusions

In this study, we determined and compared the bioactive substance contents and antioxidant capacities of differentially pigmented *C. humilis* fruits from four different varieties and explored the molecular mechanisms underlying the differences in accumulation of carotenoids and flavonoids among them. Our results show that the bioactive substance contents and antioxidant capacities in fruits of the four *C*. *humilis* varied widely. ‘Jinou No.1’ fruits had the highest antioxidant capacity, which might be due to their having the highest contents of anthocyanin, total flavonoids, and total phenols; ‘Nongda No.5’ fruits had the highest of carotenoids and highest chlorophyll contents; and ‘Nongda No.6’ fruits had the highest content of ascorbic acid. Moreover, the carotenoid contents in *C. humilis* fruits were very significantly positively correlated with the expression levels of *ChCHYB*, *ChZEP*, *ChVDE*, *ChNSY*, and several other genes, and the total flavonoid and anthocyanin contents were very significantly or significantly positively correlated with the expression levels of *ChCHS*, *ChUFGT*, and *ChFLS*. This study can provide a basis for the healthcare-oriented application of differentially pigmented *C. humilis* fruits, and can be helpful for breeding *C. humilis* varieties with higher contents of flavonoids or carotenoids.

## Figures and Tables

**Table 1:** Fruit parameters of the four different *C*. *humilis* varieties used in this study. FW: fresh weight. Different letters (^a–d^) in each line indicate significant differences at the *p* < 0.05 level.

Indexes	Jinou No.1	Nongda No.5	Nongda No.6	Nongda No.7
L*	31.66 ± 1.78 ^d^	60.64 ± 1.55 ^a^	36.36 ± 0.54 ^c^	54.44 ± 0.56 ^b^
a*	27.38 ± 0.32 ^b^	−2.22 ± 0.2 ^d^	28.53 ± 0.27 ^a^	11.4 ± 0.15 ^c^
b*	16.42 ± 0.65 ^d^	42.01 ± 2.22 ^a^	19.57 ± 1.26 ^c^	36.08 ± 1.72 ^b^
Chlorophyll content (mg/kg FW)	25.71 ± 0.28 ^b^	61.28 ± 0.40 ^a^	28.29 ± 0.44 ^ab^	23.01 ± 0.11 ^b^
Chlorophyll a Content (mg/kg FW)	10.82 ± 0.06 ^c^	23.03 ± 0.28 ^a^	11.31 ± 0.35 ^b^	8.90 ± 0.03 ^d^
Chlorophyll b Content (mg/kg FW)	14.89 ± 0.23 ^c^	38.25 ± 0.13 ^a^	16.98 ± 0.08 ^b^	14.11 ± 0.09 ^d^
Carotenoids content (mg/kg FW)	12.97 ± 0.22 ^b^	28.24 ± 0.10 ^a^	10.47 ± 1.27 ^c^	9.48 ± 0.05 ^c^
Anthocyanin content (OD·mL/100 g FW)	16.66 ± 0.29 ^a^	0.43 ± 0.10 ^d^	8.56 ± 0.18 ^b^	2.85 ± 0.23 ^c^
Total flavonoids content (mg/g FW)	0.58 ± 0.04 ^a^	0.09 ± 0.02 ^c^	0.38 ± 0.03 ^b^	0.38 ± 0.06 ^b^
Total phenols content (mg/g FW)	8.50 ± 0.38 ^a^	1.31 ± 0.21 ^b^	1.41 ± 0.42 ^b^	1.75 ± 0.77 ^b^
Ascorbic acid content (mg/100 g)	33.17 ± 2.21 ^c^	71.46 ± 2.10 ^ab^	76.10 ± 8.64 ^a^	63.35 ± 2.39 ^b^
FRAP (mg TE/kg FW)	2227.28 ± 277.55 ^a^	925.52 ± 31.64 ^b^	1226.52 ± 67.35 ^b^	1060.85 ± 96.72 ^b^
ABTS^+^ (mg TE/kg FW)	2358.95 ± 174.51 ^a^	1160.59 ± 106.11 ^b^	1232.54 ± 75.17 ^b^	1233.99 ± 5.02 ^b^
DPPH (mg TE/kg FW)	1133.83 ± 105.51 ^a^	1046.90 ± 64.60 ^ab^	950.83 ± 41.92 ^b^	979.34 ± 7.72 ^b^

**Table 2:** Correlation analysis results of the fruit parameters of the four different *C*. *humilis* varieties. Chl: Chlorophyll; Chl a: chlorophyll a; Chl b: chlorophyll b; Car: carotenoids; ABA: abscisic acid; Ant: anthocyanin; TFC: total flavonoids; TPC: total phenols; AA: ascorbic acid; FRAP: ferric reducing antioxidant power; ABTS^+^: 2,2′-azino-bis (3-ethylbenzothiazoline-6-sulfonic acid) radical cation scavenging ability; DPPH: 2,2-diphenyl-1-picrylhydrazyl free radical scavenging ability. * and ** indicate significant correlation (*p* < 0.05) and very significant correlation (*p* < 0.01), respectively.

	Chl	Chl a	Chl b	Car	Ant	TFC	TPC	AA	FRAP	ABTS^+^	DPPH
Chl	1.00 **										
Chl a	1.00 **	1.00 **									
Chl b	1.00 **	1.00 **	1.00 **								
Car	0.98 **	0.99 **	0.98 **	1.00 **							
Ant	−0.57 *	−0.52 *	−0.60 *	−0.48	1.00 **						
TFC	−0.87 **	−0.84 **	−0.88 **	−0.79 **	0.86 **	1.00 **					
TPC	−0.37	−0.32	−0.39	−0.21	0.87 **	0.76 **	1.00 **				
AA	0.38	0.34	0.40	0.22	−0.76 **	−0.70 **	−0.94 **	1.00 **			
FRAP	−0.46	−0.41	−0.49	−0.33	0.93 **	0.80 **	0.95 **	−0.88 **	1.00 **		
ABTS^+^	−0.38	−0.33	−0.41	−0.23	0.89 **	0.77 **	0.99 **	−0.93 **	0.95 **	1.00 **	
DPPH	−0.12	0.15	0.10	−0.27	0.47	0.30	0.73* *	−0.70 **	0.56 *	0.74 **	1.00 **

## Data Availability

All data are available in this article and its Supplementary Materials.

## Supplementary Materials

The following supporting information can be downloaded at: https://www.mdpi.com/article/10.3390/molecules28176272/s1, Table S1: The *C. humilis* flavonoid and carotenoid metabolism-related structural genes and their homologous genes.

## Sample Availability

Not available.
