# Supplementary material for: Comparison of Bioactive Compounds and Antioxidant Activities in Differentially Pigmented Cerasus humilis Fruits
Source: Molecules. 2023 Aug 27;28(17):6272. doi: 10.3390/molecules28176272 (PMC10488777; doi:10.3390/molecules28176272)
Supplement: Supplementary file 1 [file molecules-28-06272-s001.zip › molecules-2575291-supplementary.pdf]

Supplementary Materials

Table S1. The *C. humilis* flavonoid and carotenoid metabolism-related structural genes and their homologous genes.

C. humilis gene ID	Homologous gene ID	*C. humilis* gene name	Identity (%)	bit score
ouLi_016331-RA	AT2G37040.1	*ChPAL*	80.94	1229
ouLi_008981-RA	AT2G30490.1	*ChC4H*	72.73	421
ouLi_000029-RB	AT5G13930.1	*ChCHS*	75.44	95.1
ouLi_002001-RA	AT2G43570.1	*ChCHI*	53.20	204
ouLi_004921-RA	AT5G08640.1	*ChFLS*	60.80	431
ouLi_020095-RA	AT3G51240.1	*ChF3H*	82.51	602
ouLi_003659-RA	AT5G42800.1	*ChDFR*	74.01	530
ouLi_025349-RA	AT4G22880.1	*ChANS*	63.76	426
ouLi_010520-RA	AT5G54060.1	*ChUFGT*	57.77	250
ouLi_015020-RB	AT5G17230.3	*ChPSY*	70.59	605
ouLi_001612-RA	AT4G14210.1	*ChPDS*	81.69	965
ouLi_006033-RA	AT3G04870.1	*ChZDS*	76.21	795
ouLi_019396-RA	AT1G06820.1	*ChCRTISO*	85.25	958
ouLi_020488-RA	orange1.1g011835m.g	*ChLCYE*	93.33	94.4
ouLi_013072-RA	AT2G32640.1	*ChLCYB*	72.09	865
ouLi_015444-RA	AT5G52570.1	*ChCHYB*	67.41	414
ouLi_019786-RA	AT5G67030.1	*ChZEP*	70.99	948
ouLi_005872-RA	AT1G08550.1	*ChVDE*	49.57	387
ouLi_025636-RB	orange1.1g025796m.g	*ChNSY*	70.37	270
